# Skin and subcutaneous fascia closure at caesarean section to reduce wound complications: the closure randomised trial

**DOI:** 10.1186/s12884-020-03305-z

**Published:** 2020-10-08

**Authors:** Amanda J. Poprzeczny, Rosalie M. Grivell, Jennie Louise, Andrea R. Deussen, Jodie M. Dodd

**Affiliations:** 1 The University of Adelaide, The Robinson Research Institute, and Discipline of Obstetrics and Gynaecology, Adelaide, South Australia Australia; 2 Women’s and Babies Division, Department of Perinatal Medicine, The Women’s and Children’s Hospital, 72 King William Road, North Adelaide, Adelaide, South Australia 5006 Australia; 3 Flinders Medical Centre, Department of Obstetrics and Gynaecology, Southern Adelaide Local Health Network, Adelaide, South Australia Australia; 4 College of Medicine and Public Health, Flinders University, Adelaide, South Australia Australia; 5 The University of Adelaide, School of Public Health, Adelaide, South Australia Australia

**Keywords:** Caesarean birth, Surgical site infection, Wound closure, Wound complications, Suture techniques

## Abstract

**Background:**

Wound infection is a common complication following caesarean section. Factors influencing the risk of infection may include the suture material for skin closure, and closure of the subcutaneous fascia. We assessed the effect of skin closure with absorbable versus non-absorbable suture, and closure versus non-closure of the subcutaneous fascia on risk of wound infection following Caesarean section.

**Methods:**

Women undergoing caesarean birth at an Adelaide maternity hospital were eligible for recruitment to a randomised trial using a 2 × 2 factorial design. Women were randomised to either closure or non-closure of the subcutaneous fascia and to subcuticular skin closure with an absorbable or non-absorbable suture.

Participants were randomised to each of the two interventions into one of 4 possible groups: Group 1 - non-absorbable skin suture and non-closure of the subcutaneous fascia; Group 2 - absorbable skin suture and non-closure of the subcutaneous fascia; Group 3 - non-absorbable skin suture and closure of the subcutaneous fascia; and Group 4 - absorbable skin suture and closure of the subcutaneous fascia.

The primary outcomes were reported wound infection and wound haematoma or seroma within the first 30 days after birth.

**Results:**

A total of 851 women were recruited and randomised, with 849 women included in the analyses (Group 1: 216 women; Group 2: 212 women; Group 3: 212 women; Group 4: 211 women).

In women who underwent fascia closure, there was a statistically significant increase in risk of wound infection within 30 days post-operatively for those who had skin closure with an absorbable suture (Group 4), compared with women who had skin closure with a non-absorbable suture (Group 3) (adjusted RR 2.17; 95% CI 1.05, 4.45; *p* = 0.035). There was no significant difference in risk of wound infection for absorbable vs non-absorbable sutures in women who did not undergo fascia closure.

**Conclusion:**

The combination of subcutaneous fascia closure and skin closure with an absorbable suture may be associated with an increased risk of reported wound infection after caesarean section.

**Trial registration:**

Prospectively registered with the Australian and New Zealand Clinical Trials Registry, number ACTRN12608000143325, on the 20th March, 2008.

## Background

Caesarean section is one of the most commonly performed surgical procedures for women worldwide [1]. Rates of caesarean section vary widely globally, from as low as 5% in predominantly low-income countries, to as high as 47% in some high-income countries [2]. While the World Health Organization (WHO) has recommended a caesarean section rate no higher than 15% [3], it is acknowledged that the “optimal” caesarean section rate is not known [4, 5]. Among developed health care settings, approximately 30% of women will birth via caesarean section [6–9].

Women who birth by caesarean section have a 5 to 20 times increased risk of peri-partum infective complications, compared with women who birth vaginally [10–12]. Surgical site infections have been reported to occur in up to 12% of procedures [10, 13, 14]. Additionally, wound complications such as haematoma, seroma and dehiscence can complicate recovery after caesarean section [15], all of which may have a negative impact on postnatal maternal health and wellbeing, a woman’s ability to care for her infant, and her overall experience of the postnatal period.

Operative techniques vary widely between surgeons [16, 17]. While skin closure with staples compared with a subcuticular suture has been associated with an increased risk of postoperative wound complication in one systematic review which included observational data, [18] this difference was not observed when considering data from randomised trials only [19]. It is not known which (if any) type of skin suture is preferable [19, 20]. A systematic review of 10 trials of closure or non-closure of the subcutaneous fascia found a significant reduction in wound seroma but not in wound haematoma or infection [21]. The choice of suture material for skin closure and the decision to close the subcutaneous fascia can potentially influence the risk of wound infection and complications. There is currently insufficient evidence to guide practitioners as to the optimal technique to adopt.

We performed a 2 × 2 factorial randomised trial comparing the effects of absorbable versus non-absorbable skin suture, and closure or non-closure of the subcutaneous fascia, on wound infection and complication rates.

## Methods

### Design

Single centre 2 × 2 factorial randomised trial, allocated 1:1.

### Ethics

The trial was reviewed and approved by the Human Research Ethics Committee of the Women’s and Children’s Hospital, North Adelaide (Approval number: 1539/ 12).

### Participants

Eligible women were identified and recruited from a single tertiary metropolitan maternity hospital in Adelaide, South Australia. Women were identified in antenatal clinic, given a trial information sheet and counselled by a researcher, after which written informed consent was obtained. Women who were undergoing a caesarean section via a transverse suprapubic incision were eligible for inclusion. Women with a known lethal fetal anomaly or where the procedure was planned via a midline skin incision were excluded.

### Randomisation, allocation concealment, and blinding

Randomisation occurred at the time of decision to perform a caesarean section, by taking the next sequentially numbered treatment pack containing the skin suture material and indication of whether or not to perform subcutaneous fascia closure. The computer-generated randomisation schedule used balanced variable blocks, and was prepared by an investigator not involved with recruitment or clinical care of the participants. The randomisation schedule was stratified according to the type of caesarean section (emergency vs elective) and maternal BMI at hospital booking visit (BMI ≤ 25.0 kg/m^2^ versus > 25.0 kg/m^2^). Treatment packs were assembled according to the prepared schedule by a researcher not involved with recruitment or clinical care and were opaque and externally identical. Each treatment pack contained a sterile pack of suture material (3/0 absorbable or 3/0 non-absorbable) and a card documenting closure or non-closure of the subcutaneous fascia. The treatment pack was opened by the scrub nurse after delivery of the baby. Instructions were relayed to the operating surgeon, and the suture material opened onto the operating tray. The operating surgeon and participants were unblinded to treatment assignment after opening of the pack. Participant follow up and outcome data collection were performed by investigators not involved in the primary procedure and who were not aware of treatment allocation. Data analysis was performed following a statistical analysis plan finalised prior to analyses being conducted.

### Intervention

The two interventions were:

- Skin closure: subcuticular closure of the skin with a 3/0 absorbable (Caprosyn™) or non-absorbable (Prolene™) synthetic monofilament suture material
- Fascia closure: closure or non-closure of subcutaneous fascia, performed with 2/0 Vicryl® suture

Participants were randomised to each of the two interventions into one of 4 possible groups:

- Group 1 - non-absorbable skin suture and non-closure of the subcutaneous fascia;
- Group 2 - absorbable skin suture and non-closure of the subcutaneous fascia;
- Group 3 - non-absorbable skin suture and closure of the subcutaneous fascia; and
- Group 4 - absorbable skin suture and closure of the subcutaneous fascia.

### Participant follow-up

Wound complications were identified by asking women to complete a questionnaire by telephone. Additionally, hospital case-notes were reviewed at 30 days postpartum, to identify any diagnosis or signs or symptoms of wound complications, readmission to hospital, or presentation to the emergency department.

### Outcome

The primary outcomes were:

1. Wound infection reported in first 30 days after operation defined in accordance with CDC definition of superficial incisional surgical site infection [22], including evidence of purulent discharge; isolation of organisms following tissue or fluid culture; any of localised pain or tenderness, swelling, or redness; or the prescription of antimicrobial therapy.
2. Wound haematoma or seroma formation, defined as a collection of blood or serous fluid beneath the skin, diagnosed clinically or by ultrasound assessment, in first 30 days after operation [23].

Secondary outcomes included, emergency presentation to health care for wound management, need for surgical intervention including drainage or debridement in theatre or outpatient setting; need for hospital readmission for wound complications in first 30 days after operation (both self-report and case note review), and pain score day three post operative.

### Statistical analysis

Allowing for additive treatment effect with the two interventions in a factorial study design, a sample size of 1230 participants was determined on the basis of 80% power to detect a relative reduction of 40% in the incidence of wound infection (from 12% [24] to 7%), with two-sided alpha of 0.05, and allowing for 5% loss to follow up.

Analyses were performed on an intention to treat basis, according to treatment group allocated at randomisation, and followed the Statistical Analysis Plan. Analyses were performed using log binomial regression models for binary outcomes, and linear regression models for continuous outcomes. In the case of outcomes where the number of events was too small for the planned analysis to be performed, a Fisher’s Exact test was performed to test the hypothesis of no association between treatment group and outcome. An interaction term (suture type x fascia method) was included to test for synergistic effects (whether effect of suture type differed according to fascia method; whether effect of fascia closure differed according to suture type). Separate effect estimates were therefore derived for each treatment effect by level of the other treatment and are presented as RR (absorbable versus non-absorbable suture, and fascia closure versus non-closure) and 95% CI for binary outcomes, and differences in means (absorbable – non-absorbable, and fascia closure – nonclosure) and 95% CI for continuous outcomes. Both unadjusted and adjusted analyses were performed, with adjusted models including stratification variables (elective versus emergency caesarean section, BMI category ≤ 25 kg/m^2^ versus > 25 kg/m^2^), and baseline variables prespecified as potential confounders (maternal age, smoking status, diabetes, and previous caesarean section). All analyses were performed in Stata v16 (StataCorp, College Station, Texas, USA).

The methodology and reporting of this trial adhered to CONSORT guidelines [25].

## Results

### Participants

**Fig. 1:**
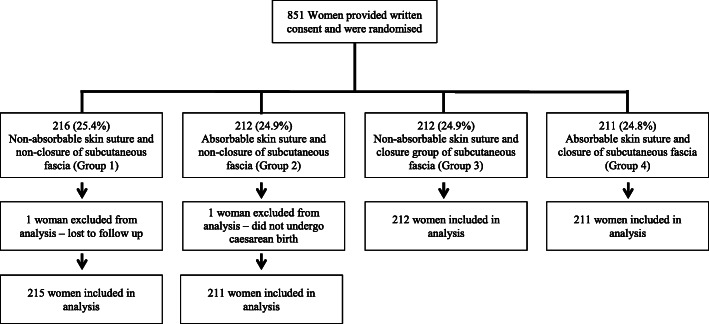
Participant flow through the Closure randomised trial

Participant flow through the trial is shown in Fig. 1, with women randomised between April 2008 and July 2011, at a metropolitan maternity unit, The Women’s and Children’s Hospital, North Adelaide, South Australia. The trial was ceased prior to achieving the estimated sample size due to difficulties in maintaining recruitment.

Overall, 851 eligible women were randomised to one of four groups: Group 1–216 women; Group 2–212 women; Group 3–212 women; and Group 4–211 women. Two women were excluded from the final analysis, one who was lost to follow up (randomised to Group 1) and one who did not undergo a caesarean birth (randomised to Group 2), leaving 849 women for whom primary outcome data was available.

Baseline demographic characteristics of women were comparable between treatment groups (Table 1). The mean age of participants was 31.48 years (SD 5.62 years), with mean early pregnancy BMI 28.60 kg/m^2^ (SD 7.36 kg/m^2^). The majority of women underwent an elective caesarean section (701 women [82.57%]), at 38.50 weeks (SD 1.85 weeks) gestation. Most women were in their second or subsequent ongoing pregnancy (656 women [77.27%]), with a previous caesarean birth (562 women [66.20%]).

**Table 1:** Baseline characteristics of women recruited to the Closure randomised trial

Characteristic	Group 1 Non-absorbable suture and non-closure fascia *N* = 215	Group 2 Absorbable suture and non-closure fascia *N* = 211	Group 3 Non-absorbable suture and closure fascia *N* = 212	Group 4 Absorbable suture and closure fascia *N* = 211	Total *N* = 849
Maternal age (years; mean (SD))	31.26 (5.73)	31.56 (5.32)	31.16 (5.81)	31.93 (5.62)	31.48 (5.62)
BMI (kg/m^2^) Median (IQR)	28.29 (7.37)	28.84 (7.60)	28.75 (7.33)	28.54 (7.18)	28.60 (7.36)
BMI category (kg/m^2^) N (%)					
- BMI ≤ 25.0	86 (40.00)	79 (37.44)	81 (38.21)	78 (36.97)	324 (38.16)
- BMI >25.0	129 (60.00)	132 (62.56)	131 (61.79)	133 (63.03)	525 (61.84)
Caesarean section type N (%)					
- elective	177 (82.33)	175 (82.94)	175 (82.55)	174 (82.46)	701 (82.57)
- emergency	38 (17.67)	36 (17.06)	37 (17.45)	37 (17.54)	148 (17.43)
Gestational age at delivery Mean (SD)	38.34 (2.02)	38.48 (2.12)	38.50 (1.85)	38.70 (1.31)	38.50 (1.85)
Ethnicity N(%)					
- Caucasian	155 (72.09)	162 (76.78)	157 (74.06)	167 (79.15)	641 (75.50)
- Other	60 (27.91)	49 (23.22)	55 (25.94)	44 (20.85)	208 (24.50)
Parity N (%)					
- 0	48 (22.33)	49 (23.22)	53 (25.00)	43 (20.38)	193 (22.73)
- 1 or more	167 (77.67)	162 (76.78)	159 (75.00)	168 (79.62)	656 (77.27)
Smoker N (%)	40 (18.60)	41 (19.43)	42 (19.81)	34 (16.11)	157 (18.49)
Singleton pregnancy N(%):	207 (96.28)	202 (95.73)	206 (97.17)	202 (95.73)	817 (96.23)
Previous Caesarean section N(%)	146 (67.91)	136 (64.45)	136 (64.15)	144 (68.25)	562 (66.20)
Previous non-caesarean laparotomy N(%)	6 (2.79)	5 (2.37)	6 (2.83)	6 (2.84)	23 (2.71)

### Wound infection and complication outcomes

Wound infection and complication outcomes by group are presented in Table 2. The overall wound infection rate was lower than expected, with 66 of 849 women (7.77%) reporting a postoperative wound infection in the 30 days postpartum (Table 2).

**Table 2:** Incidence of wound outcomes by group in the Closure randomised trial

	Group 1 Non-absorbable suture and non-closure fascia *N* = 215	Group 2 Absorbable suture and non-closure fascia *N* = 211	Group 3 Non-absorbable suture and closure fascia *N* = 212	Group 4 Absorbable suture and closure fascia *N* = 211	Total *N* = 849
Wound infection N(%)	17 (7.91)	17 (8.06)	10 (4.72)	22 (10.43)	66 (7.77)
Haematoma/Seroma N(%)	1 (0.47)	2 (0.95)	0 (0.00)	4 (1.90)	7 (0.82)
Reoperation N(%)	0 (0.00)	1 (0.47)	0 (0.00)	4 (1.90)	5 (0.59)
Readmission N(%)	0 (0.00)	0 (0.00)	1 (0.47)	2 (0.95)	3 (0.35)
Emergency presentation for wound management N(%)	14 (6.51)	8 (3.79)	9 (4.25)	17 (8.06)	48 (5.65)
Pain score at day 3 Mean (SD)	2.87 (1.91)	3.02 (1.99)	3.09 (2.06)	2.85 (2.12)	

Women who underwent fascia closure and skin closure with a non-absorbable suture (Group 3) had the lowest rate of postoperative wound infection (10 women [4.72%]), while women who underwent fascia closure and skin closure with an absorbable suture had the highest rate of postoperative wound infection (Group 4) (22 women [10.43%]) (Table 2). In women who did not have fascia closure, rates of wound infection were similar between those who had skin closure with an absorbable suture (Group 2) (17 women (7.91%)) and those who had skin closure with a non-absorbable suture (Group 1) (17 women (8.06%); see Table 2).

The occurrence of wound haematoma and seroma were very low, with only 4 women (0.47%) in the whole cohort having this complication. Two of these women (0.95%) were in Group 2 (fascia non-closure and absorbable skin suture), with a further woman each in Group 1 (0.47%) (fascia non-closure and non-absorbable skin suture) and Group 4 (0.47%) (fascia closure and absorbable skin suture) (Table 2). Only 5 women (0.59%) required reoperation, and 3 women (0.35%) readmission for wound complications in the 30 days following operation (Table 2).

### Effect estimates of different closure techniques

Effect estimates of different closure techniques are presented in Table 3. Due to the interaction term, estimates of treatment effects were derived separately for each level of the other treatment, that is:

- The effect of absorbable sutures vs non-absorbable skin suture was estimated in participants who had fascia closure, and in participants who did not have fascia closure; and
- The effect of fascia closure was estimated in participants who had absorbable vs non-absorbable skin sutures.

**Table 3:** Effect estimates by treatment group

Outcome	Unadjusted Estimate (95% CI)	Unadjusted ***p*** value	Adjusted Estimate ^**d**^ (95% CI)	Adjusted ***p*** value
**Wound Infection**		0.117*		0.119*
Non-absorbable versus absorbable suture with fascia non-closure	1.02 (0.53, 1.94)	0.954	1.01 (0.53, 1.91)	0.984
Non-absorbable versus absorbable suture with fascia closure	2.21 (1.07, 4.55)	0.031	2.17 (1.05, 4.45)	0.035
Fascia non-closure versus closure with non-absorbable suture	0.60 (0.28, 1.27)	0.181	0.59 (0.28, 1.25)	0.169
Fascia non-closure versus closure with absorbable suture	1.29 (0.71, 2.37)	0.402	1.27 (0.70, 2.31)	0.437
**Haematoma/Seroma**^**a**^				
- Fishers Exact Test		0.574		
**Re-Operation**^**a**^				
- Fishers Exact Test		0.013		
**Readmission**^**a**^				
- Fishers Exact Test		0.246		
**Emergency Presentation**		0.045*		0.046*
- Absorbable suture and fascia non-closure	0.58 (0.25, 1.36)	0.211	0.58 (0.25, 1.35)	0.205
- Absorbable suture and fascia closure	1.90 (0.87, 4.16)	0.110	1.87 (0.85, 4.08)	0.118
- Fascia closure and non-absorbable suture	0.65 (0.29, 1.47)	0.304	0.64 (0.29, 1.45)	0.288
- Fascia closure and absorbable suture	2.12 (0.94, 4.82)	0.071	2.07 (0.92, 4.69)	0.080
**Day 3 Pain Score**		0.223*		0.221*
- Absorbable suture and fascia non-closure	0.15 (−0.29, 0.59)	0.505	0.15 (−0.29, 0.59)	0.514
- Absorbable suture and fascia closure	−0.24 (−0.69, 0.21)	0.291	−0.24 (− 0.69, 0.20)	0.282
- Fascia closure and non-absorbable suture	0.22 (−0.22, 0.67)	0.327	0.23 (−0.21, 0.68)	0.309
- Fascia closure and absorbable suture	−0.17 (− 0.61, 0.27)	0.459	− 0.16 (− 0.60, 0.28)	0.477

* Denotes *p* value for test of interaction (is the effect of one treatment affected by the other treatment)

a Too few events to allow modelling. Fisher’s Exact test was performed to test for any association between group (suture type x fascia closure) and outcome

b These outcomes were assessed at two time points; planned analyses were log binomial regression model (for binary categorisation) and ordinal logistic regression (for original 3 categories), with GEE to account for repeated measures. Due to extremely small numbers in ‘dissatisfied’ category, neither of these modelling approaches was possible. Instead, Fisher’s Exact test has been performed separately at each time point

c Log Poisson regression with robust variance has been used for the adjusted model instead of log binomial regression due to convergence issues

d Adjusted analyses included stratification variables (emergency vs elective caesarean section, BMI category (≤25 vs >25), age, smoking status, diabetes and previous caesarean section

Among women who underwent fascia closure and had skin closure with an absorbable suture (Group 4), there was a statistically significant increased risk of developing a wound infection in the first 30 days following operation, compared with women who underwent fascia closure and then had skin closure with a non-absorbable suture (Group 3) (adjusted relative risk 2.17; 95% CI 1.05, 4.45; *p* = 0.035) (Table 3). Among women who did not have fascia closure, there were no statistically significant differences in the risk of wound infection among women who underwent skin closure with absorbable suture (Group 2), compared with women who underwent skin closure with non-absorbable suture (Group 1) (adjusted relative risk 1.01; 95% CI 0.53, 1.91; *p* = 0.984) (Table 3).

Similarly, among women who underwent skin closure with absorbable suture, there was no statistically significant difference in risk of wound infection in women who underwent fascia closure (Group 4), compared with women who did not have fascia closure (Group 2) (adjusted relative risk 1.27; 95% CI 0.70, 2.31; *p* = 0.437) (Table 3). In women who had skin closure with non-absorbable suture, there was very weak evidence of a lower risk of wound infection for those who had fascia closure (Group 3), compared with women who did not have fascia closure (Group 1), although the difference was not statistically significant (adjusted relative risk 0.59; 95% CI 0.28, 1.25; *p* = 0.169) (Table 3).

With regards to the outcome of wound haematoma or seroma, there were too few events to perform the planned analysis. Fisher’s Exact test showed no evidence of an association between treatment group and risk of haematoma or seroma.

While there was a statistically significant effect of wound closure technique on risk of re-operation (Fisher’s exact test, *p* = 0.013), there were too few events to perform modelling on this outcome (Table 3). Four women who required re-operation were in the fascia closure and absorbable skin suture (Group 4) (1.90%) and 1 woman in the fascia non-closure and absorbable skin suture group (Group 1) (0.47%) (Table 2).

There was no statistically significant treatment effect of any of the wound closure techniques on readmission rates, or emergency presentation for wound review (Table 3).

## Discussion

### Main findings

Overall, we found no strong evidence of effect for either the use of absorbable versus non-absorbable skin suture, or closure versus non-closure if the subcutaneous fascia. There was very weak evidence to suggest that the combination of subcutaneous fascia closure and skin closure with a non-absorbable skin suture was associated with a reduced risk of infection, but this finding should be interpreted with caution as no statistical adjustment has been made for multiple comparisons. Given low event rates and likelihood that any effect of the interventions is of small magnitude, future trials would have to be very large to properly investigate differences while accounting for any possible effect modification.

### Comparison with published literature

We have shown that there is some weak evidence to suggest that the combined intervention of subcutaneous fascia closure and skin suturing with absorbable monofilament suture (Caprosyn™), is associated with an increased risk of postoperative wound infection, in comparison with subcutaneous fascia closure and skin suturing with non-absorbable monofilament suture (Prolene™). While previous studies have shown no statistically significant difference in wound infection rates when comparing the use of absorbable versus non-absorbable skin sutures [26–28], the combination of subcutaneous fascia closure (or non-closure) with different skin sutures has not been considered.

One possible reason for our findings is that the burden of absorbable suture material is related to the increased relative risk of wound infection, among women who underwent subcutaneous fascia closure and skin closure with absorbable suture. Both the suture used for subcutaneous fascia closure (Vicryl®), and the suture used for skin closure (Caprosyn™) are absorbable. Vicryl® is a poly-filament, braided absorbable synthetic suture, which is completely absorbed within 56–70 days [29]. Caprosyn™ is a short-term absorbable monofilament suture, which is completely absorbed within 56 days [30]. Whenever a foreign body such as a suture, is placed within tissue, it induces an inflammatory reaction [31]. Absorption of absorbable suture material Vicryl® can cause tissue reactions that consist of free fluid, that may facilitate bacterial growth, [31] and the congregation of macrophages and fibroblasts, lymphocytes and plasma cells, which gradually reduce in number and concentration postoperatively [32, 33]. Optimal wound healing requires an appropriate amount of tissue inflammation [34], however it is possible that the additive burden of concurrent tissue inflammatory responses is sufficient to result in excessive inflammation, decreased efficiency of removal of contaminating micro-organisms [34], and an increased risk of clinical wound infection.

Potentially at odds with this theory is the observation that women who had skin closure with a non-absorbable suture and did not have closure of the subcutaneous fascia had rates of infection similar to women with fascia closure and absorbable skin sutures. This may be due to an additive effect of two sources of an inflammatory response to foreign material and the possibility that closure of the subcutaneous fascia is somewhat protective from post-operative wound infection, particularly in a population of women where more than 60% have a BMI of 25 kg/m^2^ or more.

It is generally acknowledged that routine closure of the subcutaneous fascia is not recommended, having not been shown to reduce the risk of wound infection (NICE guidelines 2015) [35]. Closure of fascia among women with more than two centimetres depth of subcutaneous fat to reduce wound infection, possibly secondary to elimination of wound dead space has been considered with mixed findings [36, 37].

Maternal overweight and obesity is an increasingly common issue in current obstetric practice, with approximately 50% of women across developed countries entering pregnancy overweight or obese [6, 38–40]. Overweight and obesity represents a significant independent risk factor for wound infection [41, 42], and at a population level, considering the use of non-absorbable, rather than absorbable skin sutures among women who require closure of the subcutaneous fascia may result in significant reductions in wound infection, and warrants further consideration.

### Strengths and limitations

A major strength of our study is the large sample size of 851 women randomised. To our knowledge, this represents the largest randomised trial of wound closure techniques published to date. Although we did not attain our pre-specified sample size of 1230 women due to slow recruitment, our results represent a significant addition to the published literature on the effect of different wound and subcutaneous fascia closure techniques on wound infection and complication rates. However, this study remains underpowered for the primary outcome, with a lower than anticipated rate of both wound infection and associated complications rates, and the possibility of synergistic effects between the two interventions, a much larger sample size would be required to properly study these techniques.

Our finding of a statistically significant increased relative risk of wound infection among women who had fascia closure and skin closure with an absorbable suture is supported by the direction of the effect estimate towards a lower relative risk of wound infection among women who underwent fascia closure and skin closure with a non-absorbable suture, despite this effect estimate not being statistically significant. Additionally, while there were too few events to allow for effect estimates to be calculated with regards to our secondary outcome of wound reoperation, four out of five women who underwent a wound reoperation had undergone fascia closure and skin closure with an absorbable suture, further supporting our findings.

## Conclusions

Use of non-absorbable skin suture should be considered at caesarean section, as should consideration of closure of the subcutaneous fascia. These findings may be of significant clinical relevance in light of increasing rates of maternal overweight and obesity. Further research should consider fascia closure or non-closure in women with high BMI and the appropriate skin suture material in this population.

## Data Availability

Additional trial-related documents and requests for de-identified data (aggregate or individual participant level) may be requested by written application to the corresponding author and will be considered on an individual basis by the trial authors.

## Abbreviations

BMI: Body mass index
CDC: Centre for Disease Control and Prevention
CI: Confidence interval
CONSORT: Consolidated Standards of Reporting Trials
GEE: Generalised estimating equation
IQR: Interquartile range
N: Number
NICE: National Institute for Health and Care Excellence
RR: Relative risk
SD: Standard deviation
WHO: World Health Organization
